# Cannabidiol Administered During Peri-Adolescence Prevents Behavioral Abnormalities in an Animal Model of Schizophrenia

**DOI:** 10.3389/fphar.2018.00901

**Published:** 2018-08-21

**Authors:** Fernanda F. Peres, Mariana C. Diana, Raquel Levin, Mayra A. Suiama, Valéria Almeida, Ana M. Vendramini, Camila M. Santos, Antônio W. Zuardi, Jaime E. C. Hallak, José A. Crippa, Vanessa C. Abílio

**Affiliations:** ^1^Department of Pharmacology, Federal University of São Paulo, São Paulo, Brazil; ^2^National Institute for Translational Medicine, National Council for Scientific and Technological Development, Ribeirão Preto, Brazil; ^3^Interdisciplinary Laboratory of Clinical Neurosciences, Department of Psychiatry, Federal University of São Paulo, São Paulo, Brazil; ^4^Department of Neuroscience and Behavior, University of São Paulo, Ribeirão Preto, Brazil

**Keywords:** schizophrenia, cannabidiol, SHR strain, prevention, side effects, animal models, serotonin, BDNF

## Abstract

Schizophrenia is considered a debilitating neurodevelopmental psychiatric disorder and its pharmacotherapy remains problematic without recent major advances. The development of interventions able to prevent the emergence of schizophrenia would therefore represent an enormous progress. Here, we investigated whether treatment with cannabidiol (CBD – a compound of *Cannabis sativa* that presents an antipsychotic profile in animals and humans) during peri-adolescence would prevent schizophrenia-like behavioral abnormalities in an animal model of schizophrenia: the spontaneously hypertensive rat (SHR) strain. Wistar rats and SHRs were treated with vehicle or CBD from 30 to 60 post-natal days. In experiment 1, schizophrenia-like behaviors (locomotor activity, social interaction, prepulse inhibition of startle and contextual fear conditioning) were assessed on post-natal day 90. Side effects commonly associated with antipsychotic treatment were also evaluated: body weight gain and catalepsy throughout the treatment, and oral dyskinesia 48 h after treatment interruption and on post-natal day 90. In experiment 2, serum levels of triglycerides and glycemia were assessed on post-natal day 61. In experiment 3, levels of BDNF, monoamines, and their metabolites were evaluated on post-natal days 61 and 90 in the prefrontal cortex and striatum. Treatment with CBD prevented the emergence of SHRs’ hyperlocomotor activity (a model for the positive symptoms of schizophrenia) and deficits in prepulse inhibition of startle and contextual fear conditioning (cognitive impairments). CBD did not induce any of the potential motor or metabolic side effects evaluated. Treatment with CBD increased the prefrontal cortex 5-HIAA/serotonin ratio and the levels of 5-HIAA on post-natal days 61 and 90, respectively. Our data provide pre-clinical evidence for a safe and beneficial effect of peripubertal and treatment with CBD on preventing positive and cognitive symptoms of schizophrenia, and suggest the involvement of the serotoninergic system on this effect.

## Introduction

Schizophrenia is a debilitating psychiatric disorder whose pharmacotherapy remains unable to treat all core symptoms. In addition, currently available treatments are associated with high rates of treatment resistance and significant motor and metabolic side effects (Briles et al., 2012). The onset of schizophrenia occurs mainly between late adolescence and early adulthood and it is preceded by a prodromal phase. Individuals with prodromal signs or recent functional decline associated with genetic risk are considered at ultra-high risk for developing schizophrenia (Gee and Cannon, 2011). It has been suggested that pharmacological intervention during the prodromal stage might retard or even detain the emergence of full-blown psychosis (Piras et al., 2014). In 2010, researchers predicted that prevention strategies would be implemented on the following decades and could diminish first-episode psychosis (Insel, 2010; Tandon et al., 2010). Nonetheless, so far few studies have investigated potential preventive pharmacological interventions (Lambert et al., 2016). It is also noteworthy that only 30–40% of ultra-high risk individuals convert to full psychotic state (Gee and Cannon, 2011). Therefore, preventive strategies must be effective on halting schizophrenia symptoms and also safe for the ultra-high risk individuals who will not convert. In this scenario, pre-clinical researches play a fundamental role in the development of more effective and non-detrimental strategies.

The spontaneously hypertensive rat (SHR) strain is suggested as an animal model to study several aspects of schizophrenia. When compared with Wistar rats (WRs), SHRs display schizophrenia-like behavioral abnormalities that are reversed by antipsychotic drugs and potentiated by pro-psychotic manipulations (Calzavara et al., 2009; Calzavara et al., 2011; Levin et al., 2011). Adult SHRs display diminished social interaction (that mimics the negative symptoms of schizophrenia – File and Seth, 2003), increased locomotor activity (a model for the positive symptoms – Lipska and Weinberger, 2000), deficit in contextual fear conditioning (that reflects alterations in emotional processing – Maren et al., 2013), and impaired prepulse inhibition of startle (PPI, associated with deficits in the processing of pre-attentional information commonly seen in schizophrenia – Braff et al., 2001). In addition, we have recently performed a behavioral characterization of young (30–50 days old) SHRs. Contrary to what seen in adulthood, 30-days old SHRs do not display hyperlocomotion or PPI deficit; on the other hand, 30- and 45-days old SHRs already display decreased social interaction and impaired fear conditioning, respectively (Niigaki et al., unpublished). This early emergence of negative-like symptoms and late emergence of positive-like symptoms is in accordance with schizophrenia’s clinical course (Piskulic et al., 2012; Fusar-Poli et al., 2013). Taken as a whole, these data reinforce the SHR strain as a model to investigate the development of the disorder and not only therapeutic interventions (Levin et al., 2012, 2014; Almeida et al., 2013, 2014), but also preventive strategies (Santos et al., 2016; Diana et al., 2018).

Cannabidiol (CBD) is a non-psychotomimetic compound of *Cannabis sativa* that presents antipsychotic profile in rodents and humans (Rohleder et al., 2016). In animals, treatment with CBD attenuates several schizophrenia-like behavioral abnormalities. Acute administration of CBD restores SHRs’ impairments in both PPI and fear conditioning tasks (Levin et al., 2012, 2014). More recently, Osborne et al. (2017) described beneficial effects of prolonged treatment with CBD during late adolescence/adulthood on social interaction and memory impairments in a neurodevelopmental animal model of schizophrenia – the maternal immune activation by poly I:C (Meyer and Feldon, 2012). Accordingly, clinical studies show that prolonged treatment with CBD is able to treat the symptoms of schizophrenia patients (Rohleder et al., 2016), and the psychotic symptoms induced by L-DOPA in patients with Parkinson’s disease (Zuardi et al., 2009). Moreover, CBD does not promote motor effects, such as catalepsy and oral dyskinesia (Zuardi et al., 1991; Dos-Santos-Pereira et al., 2016), extrapyramidal side effects commonly induced by antipsychotic drugs (Briles et al., 2012). However, despite the growing evidence of CBD’s neuroprotective effects (Campos et al., 2017) and therapeutic applications in schizophrenia, so far only one previous study investigated peripubertal administration of CBD as a preventive strategy (Peres et al., 2016a): CBD prevents the emergence of hyperlocomotion in adulthood in the poly I:C model.

The aim of the present study was to evaluate whether CBD would prevent the emergence of schizophrenia-like behavioral abnormalities in the SHR model. For that, animals were treated during peri-adolescence and their behaviors were evaluated at adulthood. Throughout treatment, we assessed motor and metabolic side effects commonly associated with antipsychotic drugs. With respect to possible mechanisms of action, several molecular targets have been associated to CBD’s beneficial effects (Ibeas Bih et al., 2015). Considering its antipsychotic profile and the neurodevelopmental course of schizophrenia, we quantified the levels of dopamine, serotonin and their metabolites as well as of brain derived neurotrophic factor (BDNF) in striatum and prefrontal cortex, two regions associated to the pathophysiology of the disorder. These evaluations were performed after the end of CBD treatment and in adulthood.

## Materials and Methods

### Animals

Male WRs and SHRs from our own colony were used. Animals were maintained in groups of five in Plexiglas cages (41 cm × 34 cm × 16.5 cm) under controlled environmental conditions (22–23°C, light/dark cycle: lights on 6:30 a.m. to 6:30 p.m.) with free access to food and water. This study was approved by the Ethics Committee of Federal University of São Paulo (N 1728/11 and 3120151215). Procedures followed the guidelines of the Committee on Care and Use of Laboratory Animal Resources, National Research Council, United States.

### Drugs and Experimental Design

Cannabidiol (STI-Pharm/THC-Pharm, Frankfurt, Germany) was daily diluted in vehicle (VEH – 1% Tween 80 and saline). CBD and its vehicle were administered intraperitoneally, in a volume of 1.0 ml/kg. Before all experiments, all animals were drug and test naïve.

**FIGURE 1:**
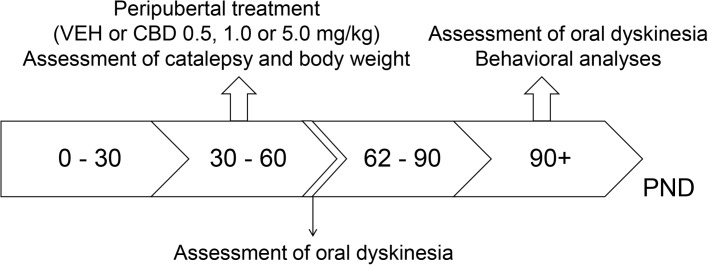
Experimental design used in experiment 1 to evaluate the preventive effects of cannabidiol (CBD) on the spontaneously hypertensive rat (SHR) strain. Wistar rats (WRs) and SHRs (*n* = 10/group) were treated from post-natal day (PND) 30 to 60 with CBD (0.5, 1.0, or 5.0 mg/kg) or vehicle (VEH). Body weight gain and catalepsy were assessed throughout the treatment. On PND 62, 48 h after treatment interruption, oral dyskinesia was assessed. Schizophrenia-like behaviors and oral dyskinesia were evaluated in adulthood (PND 90–150).

In experiment 1, WRs and SHRs were treated with VEH or 0.5, 1, or 5 mg/kg CBD (*n* = 10 per strain and drug treatment) from 30 to 60 post-natal day (PND). Body weight gain and catalepsy were regularly assessed throughout treatment. Forty-eight hours after treatment cessation (PND 62), oral dyskinesia was evaluated. On PND 90, schizophrenia-like behaviors were assessed (in the following order: locomotor activity, social interaction, prepulse inhibition of startle and contextual fear conditioning) and a second analysis of oral dyskinesia was performed (**Figure 1**).

In experiment 2, WRs and SHRs were treated with VEH or 0.5, 1 or 5 mg/kg CBD (*n* = 10 per strain and drug treatment) from 30 to 60 PND. On PND 61, 24 h after the last injection of CBD or vehicle, animals’ glycemia and serum levels of triglycerides were analyzed.

Cannabidiol doses were chosen based on previous studies from our group showing preventive and antipsychotic effects of CBD (Levin et al., 2012; Peres et al., 2016a). Sample size was calculated in accordance with the suggestion of Charan and Kantharia (2013). The calculation followed the equation: $n=[2^{*}{\text{SD}}^{2*}{\left({\text{Z}}^{\beta }\right)}^{2}]/{\text{d}}^{2}$, where SD = standard deviation, d = effect size (i.e., difference between mean values), and Z^α/2^ and Z^β^ are equal to 1.96 and 0.842, respectively, for α = 0.05 and power = 80%. Based on our previous data, we considered *SD* = 24%, and *d* = 30%.

In experiment 3, WRs and SHRs were treated with VEH or 0.5 mg/kg CBD (*n* = 5–6 per strain and drug treatment) from 30 to 60 PND. On PND 61 (24 h after the last injection of CBD or vehicle) and on PND 90 animals were decapitated and their brains were collected to the quantification of monoamines, their metabolites, and the levels of BDNF. We chose to treat animals only with the 0.5 mg/kg dose of CBD based on the results from experiment 1: CBD 0.5 mg/kg was the only dose able to modify SHRs behavior. Moreover, for the calculation of sample size, we considered *SD* = 25% and *d* = 45% (based on our previous data on neurochemical evaluations), and this allowed the reduction of sample size. Both decisions aimed to diminish the number of animals used.

### Schizophrenia-Like Behavioral Analyses

Behavioral analyses began 1 month after cessation of peripubertal treatment, on PND 90. In all behavioral assessments, the behaviors were recorded and analyzed by trained observers – previously submitted to evaluation of concordance – that were blind to rats’ experimental condition.

#### Locomotor Activity

Locomotor activity was assessed on a white and circular open-field arena (97 cm in diameter and 32.5 cm high, with an open top and a floor divided into 19 similar quadrants). The animals were individually placed in the center of the apparatus and locomotor activity was quantified during 5 min. Locomotor activity was assessed by the number of floor squares entered with all the four paws. The same protocol has already been used to assess SHRs locomotion (Calzavara et al., 2011).

#### Social Interaction

The assessment of social interaction was conducted in the same open-field arena used to evaluate locomotor activity (described above). Pairs of unfamiliar rats from the same experimental condition were placed simultaneously in the apparatus, approximately 80 cm apart. Social behavior was quantified during 10 min. Social interaction was considered when animals sniffed or followed the other animal (active social interaction) or when they lied next to each other (passive social interaction). Time spent in active and passive social interaction were scored and summed for each rat. The same protocol has already been employed to evaluate SHRs social interaction (Calzavara et al., 2011).

#### Prepulse Inhibition of Startle

PPI was assessed using a stabilimeter cage, which consisted on a wire-mesh cage (16.5 cm × 5.1 cm × 7.6 cm) suspended in a polyvinyl chloride frame (25 cm × 9 cm × 9 cm) and attached to the response platform with four thumbnail-screws. The stabilimeter and platform were positioned inside a ventilated plywood sound attenuating chamber (64 cm × 60 cm × 40 cm). The floor of the stabilimeter consisted on six stainless steel bars 3.0 mm in diameter and spaced 1.5 cm apart. Rats’ startle reaction generated a pressure on the response platform, and analog signals were analyzed by a software (Insight, São Paulo, Brazil). Two loudspeakers located 10 cm above the floor, on each lateral side of the acoustic isolation chamber, were employed to deliver all the sonorous stimuli. Calibration procedures were conducted before the experiments to ensure equivalent sensitivities of the response platforms over the test period.

For the assessment of PPI, rats were individually placed in the stabilimeter cage. After an acclimatization period (5 min exposed to a background noise of 65 dB), rats were submitted to a series of 10 stimuli (pulse alone – 120 dB, 50 ms duration), with an average inter-trial interval of 20 s. The purpose of this phase was to allow within-session habituation to the startle stimulus, and it was not included in the calculation of PPI. Thereafter, the prepulse modulation of the acoustic startle reflex was assessed. Pseudo-randomly trials divided into four different categories were presented with an average inter-trial interval of 20 s: 20 presentations of pulse alone (120 dB, 50 ms duration), 8 presentations of each prepulse alone (70, 75 and 80 dB, 3,000 Hz frequency, 20 ms duration), 10 presentations of each prepulse + pulse (with 100 ms interval), and 8 presentations of no stimulus. The level of PPI was determined by expressing the prepulse followed by pulse (PP) startle amplitude as a percentage decrease from pulse-alone (P) startle amplitude (% PPI = 100 - [100 × (PP/P)]). The same protocol has been used to assess SHRs PPI (Levin et al., 2014).

#### Contextual Fear Conditioning

The contextual fear conditioning task was performed in a dark chamber with a grid floor (22 cm × 22 cm × 22 cm – Insight, São Paulo, Brazil) and consisted on two consecutive days. On the first day (training session), the animals were individually placed in the chamber, and after 140 s, 0.4 mA foot shocks lasting 5 s were applied every 30 s, until complete the 5 min of the session. Twenty-four hours later (testing session), each animal was placed in the same dark chamber, without receiving foot shocks, during 5 min. Animal’s behavior was recorded by a video camera and the assessment was conducted over a screen. The duration of freezing behavior (complete immobility of the animal with the absence of vibrissae movements and sniffing) was scored live in testing session. The same protocol has been used to evaluate SHRs contextual fear conditioning (Calzavara et al., 2009).

### Assessment of Potential Side Effects

#### Body Weight Gain

Body weight was assessed during treatment every 4–5 days since treatment day 1.

#### Catalepsy

Catalepsy was assessed by placing each animal’s forepaws on a horizontal bar elevated 4.5 cm from the workbench. The amount of time the animal remained in the same imposed position was scored live until a maximum of 180 s. Three trials were carried out for each animal and the mean value was calculated.

Catalepsy was assessed before treatment and weekly 30 min and 24 h after CBD injection.

#### Oral Dyskinesia

Oral dyskinesia was evaluated 48 h and 1-month after cessation of the peripubertal treatment (PND 62 and 90). Animals were placed in wired cages (40 cm × 40.5 × 20 cm). Mirrors were placed behind the back and under the bottom to allow behavioral quantification when the animal faced away from the observer. Vacuous chewing movements (mouth openings in the vertical plane not directed toward physical material) and tongue protrusions were quantified live during 10 min.

#### Glycemia and Serum Levels of Triglycerides

Blood was collected from tail vein. The glucose meter One Touch (Johnson & Johnson) was employed to measure glycemia. To the evaluation of triglycerides levels, blood with EDTA was centrifuged at 4°C, 4,000 rpm, for 10 min. The serum was analyzed by the colorimetric kit Triglycerides Liquiform (LabTest).

### Neurochemical Evaluations

Animals were decapitated after anesthesia with ketamine (90 mg/kg) and xylazine (10 mg/kg), and their brains were collected for the dissection of prefrontal cortex and striatum. The brain structures were conditioned in plastic micro tubes with cap and immediately stored at -80°C, until use. Structures were unfrozen in room temperature prior to the neurochemical quantifications.

#### Quantification of Dopamine and Serotonin Levels

The tissue levels of dopamine, serotonin and their metabolites 3,4-dihydroxyphenylacetic acid (DOPAC), homovanillic acid (HVA) and 5-hydroxyindoleacetic acid (5-HIAA) were measured using a high-performance liquid chromatography (HPLC) with electrochemical detection. The tissue samples were homogenized in a solution of 0.1 M perchloric acid and spiked with 1 ng of isoproterenol per 1 mg of wet tissue (internal standard), then centrifuged at 14,000 rpm for 15 min at 4°C. The supernatant was filtered with a 0.45 μm membrane, its pH was adjusted to 3 with 1 M sodium acetate solution, and injected into the HPLC system. The chromatography system consisted of an ECD-700 electrochemical detector with an electrolysis cell (Eicom, United States), an EP-700 pump (Eicom, United States), and an Octadecylsilyl SC-3ODS column (3 μm, 100 mm × 3 mm, Eicom). The mobile phase consisted of 80% 0.1 M citrate-acetate buffer (pH 3.5), 20% methanol, 220 mg/l sodium octane sulfonate (SOS) and 5 mg/l EDTA-2Na. The flow rate was 340 μl/min. The electrode potential was set at 0.75 V. Eicom Envision EPC-700 version 1.0b was used for data collection and analysis. Response ratio (area under curve for the monoamine/area under curve for the internal standard) was calculated for each neurotransmitter. The ratio between dopamine and its metabolites [(DOPAC+HVA)/dopamine] was used as an indicator of dopamine turnover, and the ratio 5-HIAA/serotonin was used as an indicator of serotonin turnover.

#### Quantification of BDNF Levels

The tissue levels of BDNF were measured using an ELISA system. The tissue samples were homogenized in a lysis buffer with protease inhibitor (Sigma^®^), centrifuged at 14,000 rpm for 15 min at 4°C, and the supernatant was collected. The total amount of protein was determined for each animal by Bradford assay (Bio-Rad^®^). For the quantification of BDNF levels 400 μg of protein from each sample was used. The samples were analyzed by BDNF Emax^®^ ImmunoAssay System (Promega), following the manufacturer’s instructions.

### Statistical Analysis

Levene’s and Shapiro–Wilks tests were used to determine whether the data were parametric. When non-parametric (social interaction and contextual fear conditioning), data were logarithmically transformed to allow a parametric analyzes.

Data from oral dyskinesia, locomotor activity, social interaction, contextual fear conditioning, glycemia, triglycerides, BDNF, and monoamines levels were analyzed by two-way ANOVA (strain × treatment) followed by Duncan’s test. Data from PPI were analyzed by repeated measures three-way ANOVA (strain × treatment × prepulse intensity) followed by two-way ANOVA (strain × treatment) and by Duncan’s test. Body weight gain and catalepsy data were analyzed by repeated measures three-way ANOVA (strain × treatment × time) followed by two-way ANOVA (strain × treatment) and by Duncan’s test. The *p* < 0.05 was used as a criterion for statistical significance. Statistical analyzes were conducted on the software SPSS 20.

## Results

### Schizophrenia-Like Behavioral Analyses

#### Locomotor Activity

**FIGURE 2:**
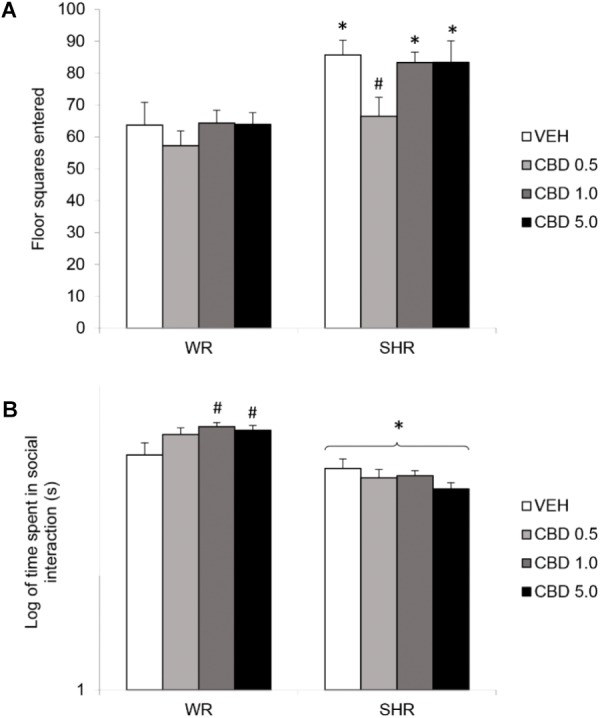
Locomotor activity, assessed by the number of floor squares entered **(A)**, and social interaction duration **(B)** of adult WRs and SHRs (*n* = 10/group) treated with vehicle (VEH) or cannabidiol (CBD – 0.5 mg/kg, 1.0 mg/kg, or 5.0 mg/kg) during peri-adolescence (30–60 post-natal day). Data from social interaction duration(s) were logarithmically transformed. Data reported as mean ± SEM. Two-way ANOVA followed by Duncan’s test. ^∗^*p* < 0.001 compared to WRs of same treatment. ^#^*p* < 0.05 compared to VEH-treated animals of the same strain. SHR, spontaneously hypertensive rats; WR, Wistar rats.

Two-way ANOVA detected a significant effect of strain [*F*(1,73) = 22.781; *p* < 0.001] and treatment [*F*(1,73) = 2.801; *p* = 0.046]. SHRs displayed increased locomotor activity. Although there was no interaction effect, *post hoc* analysis revealed that only SHRs treated with 0.5 mg/kg CBD displayed decreased locomotor activity when compared to VEH-treated SHRs (without differing from WRs). Therefore, treatment with CBD 0.5 mg/kg prevented the development of hyperlocomotion in SHRs (**Figure 2A**).

#### Social Interaction

Two-way ANOVA showed a significant interaction between strain and treatment [*F*(1,72) = 3.324; *p* = 0.024], and an effect of strain [*F*(1,72) = 59.249; *p* < 0.001]. *Post hoc* analysis revealed that SHRs displayed diminished social interaction that was not reversed by CBD. Administration of CBD 1.0 or 5.0 mg/kg increased WRs’ total social interaction (**Figure 2B**). Means and standard errors are depicted in **Table 1**.

**Table 1:** Social interaction and freezing behavior on fear conditioning of WRs and SHRs.

	WR	SHR
**Total social interaction(s)**		
VEH	142.30 ± 21.73	111.10 ± 15.64
CBD 0.5	181.40 ± 20.99	94.90 ± 11.41
CBD 1.0	199.30 ± 13.36	93.90 ± 6.22
CBD 5.0	189.50 ± 15.30	79.00 ± 7.22
**Freezing behavior on fear conditioning(s)**		
VEH	79.50 ± 14.47	48.25 ± 7.47
CBD 0.5	124.25 ± 18.07	79.00 ± 9.29
CBD 1.0	130.33 ± 14.92	52.00 ± 5.16
CBD 5.0	95.63 ± 19.19	42.88 ± 2.95

*CBD, cannabidiol; PND, post-natal day; SHR, spontaneously hypertensive rats; VEH, vehicle; WR, Wistar rats. WRs and SHRs were treated with vehicle (VEH) or cannabidiol (CBD – 0.5 mg/kg, 1.0 mg/kg, or 5.0 mg/kg) during peri-adolescence (30–60 post-natal day). Social interaction time(s) and freezing behavior on contextual fear conditioning(s) were assessed on adulthood (PND +90). Data reported as mean ± SEM.*

#### Prepulse Inhibition of Startle

**FIGURE 3:**
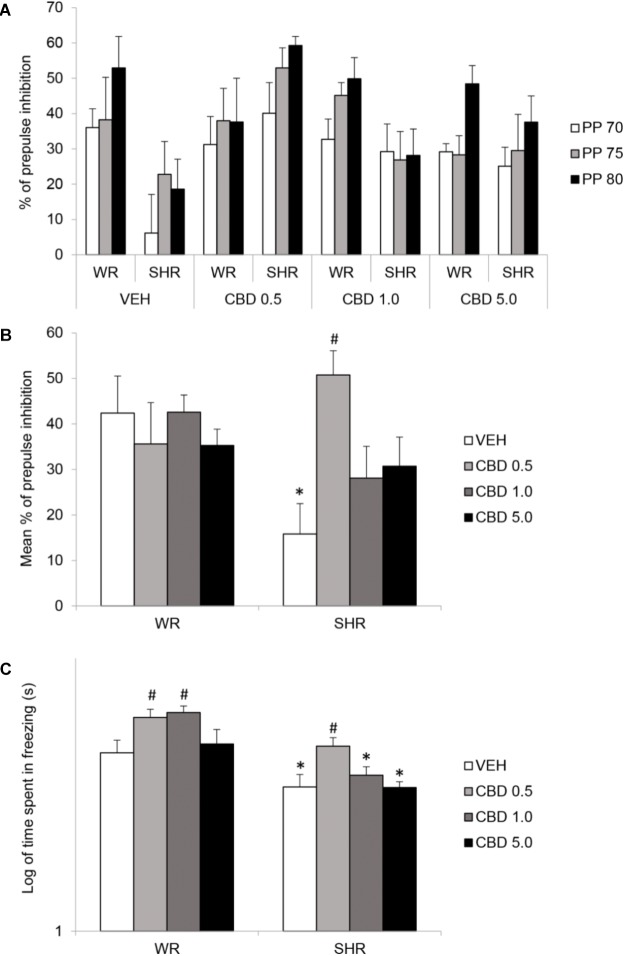
Percentage of prepulse inhibition of startle for each prepulse (PP) intensity **(A)**, mean percentage of prepulse inhibition of startle for all intensities **(B)**, and freezing response during contextual fear conditioning test **(C)** of adult WRs and SHRs (*n* = 10/group) treated with vehicle (VEH) or cannabidiol (CBD – 0.5 mg/kg, 1.0 mg/kg, or 5.0 mg/kg) during peri-adolescence (30–60 post-natal day). Data from freezing duration(s) in contextual fear conditioning were logarithmically transformed. Data reported as mean ± SEM. Two-way ANOVA followed by Duncan’s test. ^∗^*p* < 0.001 compared to WRs of same treatment. ^#^*p* < 0.05 compared to VEH-treated animals of the same strain. SHR, spontaneously hypertensive rats; WR, Wistar rats.

Repeated measures three-way ANOVA detected a significant interaction between strain and treatment [*F*(3,72) = 3.688; *p* = 0.016], and an effect of prepulse intensity [*F*(2,144) = 11,804; *p* < 0,001]. *Post hoc* analysis of the PPI mean showed that SHRs treated with VEH displayed diminished PPI. Treatment with CBD 0.5 mg/kg prevented the emergence of PPI impairments in SHRs (**Figures 3A,B**).

#### Contextual Fear Conditioning

Two-way ANOVA showed a significant effect of strain [*F*(1,73) = 34.627; *p* < 0.001] and treatment [*F*(3,73) = 6.385; *p* = 0.001]. SHRs displayed decreased freezing behavior. *Post hoc* analysis revealed that treatment with 0.5 mg/kg CBD increased freezing response in both strains. SHRs treated with 0.5 mg/kg CBD did not differ from VEH-treated WRs. Therefore, treatment with CBD 0.5 mg/kg restored the decrease in contextual fear conditioning displayed by SHRs. Treatment with 1.0 mg/kg CBD increased freezing response in WRs (**Figure 3C**). Means and standard errors are depicted in **Table 1**.

### Assessment of Potential Side Effects

#### Body Weight Gain

**FIGURE 4:**
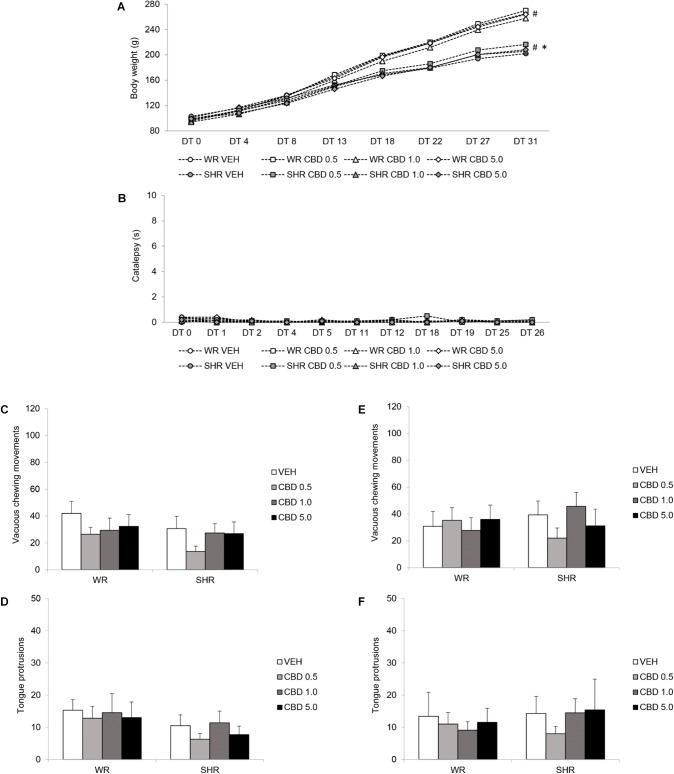
Potential side effects assessed during and after peripubertal treatment with cannabidiol (CBD). WRs and SHRs were treated with vehicle (VEH) or CBD (0.5 mg/kg, 1.0 mg/kg or 5.0 mg/kg) during peri-adolescence (30–60 post-natal day). Body weight gain in grams **(A)**, and catalepsy behavior in seconds **(B)**, were evaluated throughout treatment. Vacuous chewing movements and tongue protrusions were assessed 48 h **(C,D)** and 1 month after **(E,F)** treatment cessation (post-natal days 62 and 90, respectively). Data reported as mean ± SEM. Two-way ANOVA (vacuous chewing movements, tongue protrusions) or repeated measures three-way ANOVA (catalepsy, body weight). ^∗^*p* < 0.001 compared to WRs of same treatment, from DT13 to DT31. ^#^*p* < 0.001 compared to the same experimental group in all other days of treatment, from DT8 to DT31. DT, day of treatment; SHR, spontaneously hypertensive rats; WR, Wistar rats.

Repeated measures three-way ANOVA revealed a significant interaction between time and strain [*F*(1.856,135.468) = 120,036; *p* < 0.001], as well as effect of time [*F*(1.856,135.468) = 2906.983; *p* < 0.001] and strain [*F*(1,73) = 23.176; *p* < 0.001]. Body weight of both WRs and SHRs increased with age, and SHRs displayed lower body weight gain. Treatment with CBD did not modify animals’ body weight gain (**Figure 4A**).

#### Catalepsy

Repeated measures three-way ANOVA did not detect significant effects of time, treatment or strain. Treatment with CBD did not induce catalepsy (**Figure 4B**).

#### Oral Dyskinesia

Two-way ANOVA did not reveal significant effect of treatment or strain on vacuous chewing movements or tongue protrusions. Treatment with CBD did not induce oral dyskinesia (**Figures 4C–F**).

#### Glycemia and Triglycerides

**FIGURE 5:**
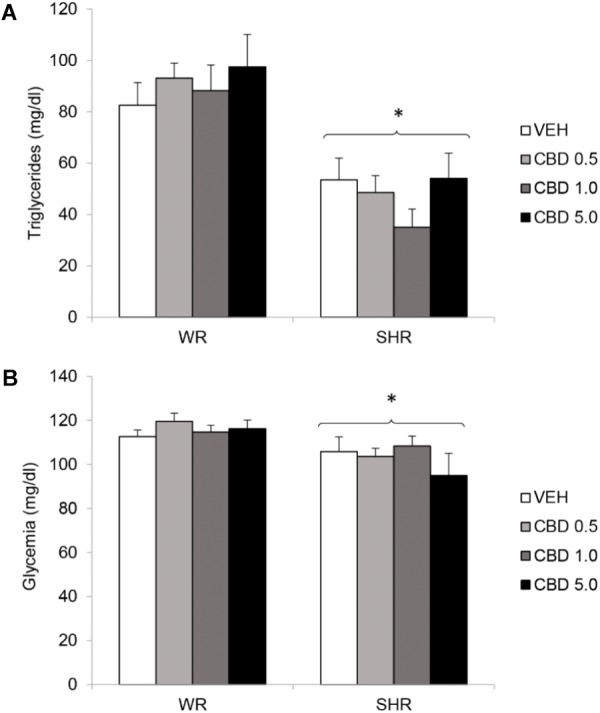
Serum levels of triglycerides **(A)** and glycemia **(B)** of adult WRs and SHRs (*n* = 10/group) treated with vehicle (VEH) or cannabidiol (CBD – 0.5 mg/kg, 1.0 mg/kg, or 5.0 mg/kg) during peri-adolescence (30–60 post-natal day). Glycemia and triglycerides were assessed in the end of treatment (post-natal day 61). Data reported as mean ± SEM. Two-way ANOVA. SHR, spontaneously hypertensive rats; WR, Wistar rats. ^∗^*p* < 0.05 compared to WRs of the same treatment.

Two-way ANOVA revealed a significant effect of strain for both glycemia [*F*(1,72) = 11.042; *p* = 0.001] and triglycerides [*F*(1,72) = 44.138; *p* < 0.001]. There was no effect of treatment or interaction between treatment and strain. SHRs displayed lower levels of triglycerides and glycemia. Treatment with CBD did not modify these metabolic parameters (**Figure 5**).

### Neurochemical Evaluations

#### Quantification of Dopamine and Serotonin Levels

**FIGURE 6:**
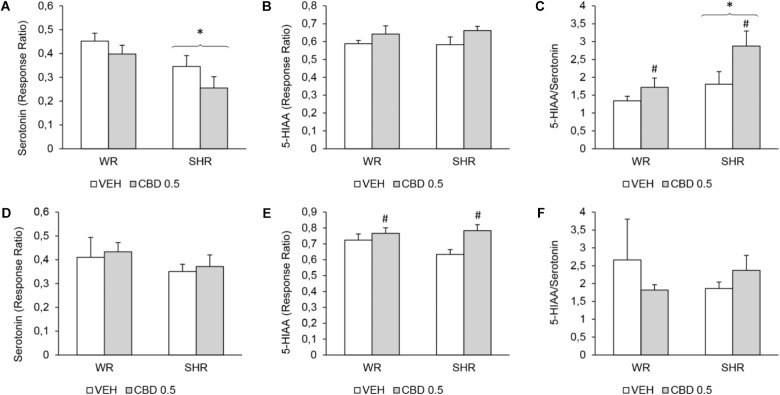
Levels of serotonin, 5-hydroxyindoleacetic acid (5-HIAA), and 5-HIAA/serotonin ratio on the prefrontal cortex of WRs and SHRs (*n* = 5–6/group) treated with vehicle (VEH) or 0.5 mg/kg cannabidiol (CBD 0.5) during peri-adolescence (30–60 post-natal day). The levels of monoamines were assessed on post-natal day 61 **(A–C)** and on post-natal day 90 **(D–F)**. Data expressed as response ratio: area under curve for the monoamine/area under curve for the internal standard. Data reported as mean ± SEM. Two-way ANOVA. ^∗^*p* < 0.001 compared to WRs of same treatment. ^#^*p* < 0.05 compared to VEH-treated animals of the same strain. SHR, spontaneously hypertensive rats; WR, Wistar rats.

The results of the quantification of dopamine, serotonin, and their metabolites are summarized on **Figure 6** and on **Tables 2**, **3**.

**Table 2:** Levels of serotonin and its metabolite on the striatum of WRs and SHRs, on PND 61 and on PND 90.

	Serotonin	5-HIAA	5-HIAA/Serotonin
**PND 61**			
**Striatum**			
WR-VEH	0.1301 ± 0.0195	1.1370 ± 0.0674	9.5194 ± 1.2841
WR-CBD 0.5	0.1299 ± 0.033	1.1558 ± 0.0299	11.1962 ± 1.9660
SHR-VEH	0.1422 ± 0.0182	1.1614 ± 0.0301	8.6637 ± 1.0678
SHR-CBD 0.5	0.1025 ± 0.0092	1.1573 ± 0.0901	11.8575 ± 1.7045
**PND 90**			
**Striatum**			
WR-VEH	0.5455 ± 0.0488	1.1027 ± 0.0735	27.2991 ± 1.2514
WR-CBD 0.5	0.4473 ± 0.0616	1.1095 ± 0.0706	24.8283 ± 1.5249
SHR-VEH	0.5680 ± 0.0497	1.0829 ± 0.0683	27.1052 ± 1.6321
SHR-CBD 0.5	0.5261 ± 0.0717	1.1014 ± 0.0931	27.5118 ± 1.2357

*5-HIAA, 5-hydroxyindoleacetic acid; CBD, cannabidiol; PND, post-natal day; SHR, spontaneously hypertensive rats; VEH, vehicle; WR, Wistar rats. WRs and SHRs were treated with vehicle (VEH) or 0.5 mg/kg cannabidiol (CBD 0.5) during peri-adolescence (30–60 post-natal day). Serotonin and its metabolite (5-HIAA) levels were assessed on PND 61 and 90. Data shown as response ratio: area under curve for the monoamine/area under curve for the internal standard. Data reported as mean ± SEM. Two-way ANOVA.*

**Table 3:** Levels of dopamine and its metabolites on the prefrontal cortex and striatum of WRs and SHRs, on PND 61 and on PND 90.

	Dopamine	(DOPAC+HVA)/Dopamine
**PND 61**		
**Prefrontal cortex**		
WR-VEH	0.1441 ± 0.0045	0.6987 ± 0.0448
WR-CBD 0.5	0.1570 ± 0.0103	0.6476 ± 0.0633
SHR-VEH	0.1407 ± 0.0135	0.7552 ± 0.0669
SHR-CBD 0.5	0.1516 ± 0.0050	0.7258 ± 0.0801
**Striatum**		
WR-VEH	2.9010 ± 0.6246	7.2095 ± 1.3509
WR-CBD 0.5	2.5241 ± 0.1833	6.3722 ± 0.5831
SHR-VEH	2.9828 ± 0.2737	5.7081 ± 0.9843
SHR-CBD 0.5	2.4877 ± 0.1534	6.5346 ± 0.6108
**PND 90**		
**Prefrontal cortex**		
WR-VEH	0.1383 ± 0.0172	0.9884 ± 0.1450
WR-CBD 0.5	0.1424 ± 0.0077	0.9943 ± 0.1005
SHR-VEH	0.1695 ± 0.0061^*^	1.0442 ± 0.0418
SHR-CBD 0.5	0.1682 ± 0.0090^*^	1.1458 ± 0.0516
**Striatum**		
WR-VEH	13.7074 ± 1.3901	1.0124 ± 0.1511
WR-CBD 0.5	10.9551 ± 1.3598	1.3086 ± 0.1496
SHR-VEH	12.8592 ± 1.4039	1.1571 ± 0.1764
SHR-CBD 0.5	12.8431 ± 1.3031	1.2330 ± 0.2757

*CBD, cannabidiol; DOPAC, 3,4-dihydroxyphenylacetic acid; HVA, homovanillic acid; PND, post-natal day; SHR, spontaneously hypertensive rats; VEH, vehicle; WR, Wistar rats. WRs and SHRs were treated with vehicle (VEH) or 0.5 mg/kg cannabidiol (CBD 0.5) during peri-adolescence (30–60 post-natal day). Dopamine and its metabolites (DOPAC and HVA) levels were assessed on PND 61 and 90. Data shown as response ratio: area under curve for the monoamine/area under curve for the internal standard. Data reported as mean ± SEM. Two-way ANOVA. ^∗^*p* < 0.05 compared to WRs.*

In the prefrontal cortex, on PND 61, SHRs displayed decreased levels of serotonin [*F*(1,17) = 9.465; *p* = 0.007] and increased 5-HIAA/serotonin ratio [*F*(1,17) = 7.756; *p* = 0.013]. Treatment with CBD increased 5-HIAA/serotonin ratio in both strains [*F*(1,17) = 6.123; *p* = 0.024]. On PND 90, SHRs displayed increased levels of dopamine [*F*(1,17) = 7.321; *p* = 0.014] and treatment with CBD increased the levels of 5-HIAA [*F*(1,17) = 8.786; *p* = 0.008].

In striatum, no effect of strain or treatment was detected.

#### Quantification of BDNF Levels

**FIGURE 7:**
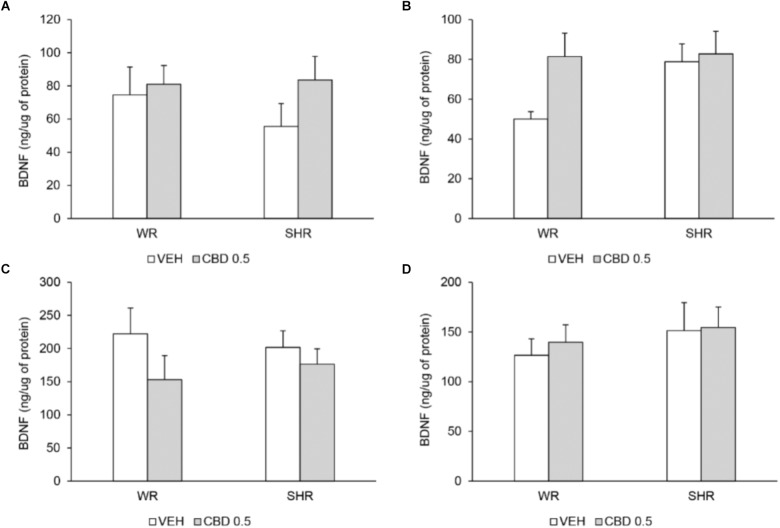
BDNF levels (ng of BDNF/μg of protein) in prefrontal cortex **(A)** and striatum **(B)** on post-natal day 61, and in prefrontal cortex **(C)** and striatum **(D)** on post-natal day 90 of WRs and SHRs (*n* = 5/group) treated with vehicle (VEH) or 0.5 mg/kg cannabidiol (CBD 0.5) during peri-adolescence (30–60 post-natal day). Data reported as mean ± SEM. Two-way ANOVA.

Two-way ANOVA did not detect significant effects of strain or treatment. The BDNF levels in prefrontal cortex and striatum did not differ between the strains and were not modified by treatment with CBD (**Figure 7**).

## Discussion

Implementing preventive strategies would represent a major improvement in schizophrenia outcomes. Given that only 30–40% of ultra-high risk individuals convert to full psychotic state (Gee and Cannon, 2011), the scarce clinical investigations of preventive treatments face important ethical and technical challenges (Lambert et al., 2016). Pre-clinical studies, therefore, play a fundamental role, since they allow investigating early interventions with the clear distinction between subjects who will develop behavioral abnormalities from those who will not. Nonetheless, few pre-clinical studies have addressed this important clinical issue, with the limitation of not evaluating potential side effects or focusing only on psychosis-like behaviors (Piontkewitz et al., 2009, 2011; Meyer et al., 2010; Herrmann et al., 2014; Peres et al., 2016a).

In this scenario, CBD emerges as a promising intervention: in addition to displaying an antipsychotic profile, CBD does not induce the motor side effects commonly associated with the use of antipsychotic drugs (Zuardi et al., 1991; Leweke et al., 2012; Dos-Santos-Pereira et al., 2016). On contrary, CBD can attenuate the development of parkinsonian and/or dyskinetic motor behaviors induced by several agents (El-Alfy et al., 2010; Gomes et al., 2013; Peres et al., 2016b; Sonego et al., 2016). Supported by these data, we first investigated CBD’s preventive potential in the poly I:C neurodevelopmental model of schizophrenia. We presented, for the first time, evidence of CBD’s preventive action (Peres et al., 2016a). Nevertheless, we were able to evaluate CBD’s effect only on hyperlocomotion. Here, using the SHR strain, we were able to confirm CBD’s beneficial effect on hyperlocomotion and extend it to other schizophrenia-like behavioral abnormalities: the deficits in PPI and in contextual fear conditioning.

Interestingly, SHRs – mainly young animals – have been suggested as an animal model to study attention deficit/ hyperactivity disorder (ADHD), based on theirs impulsivity and hyperactivity (Sagvolden et al., 2005). Nonetheless, in these studies, authors use Wistar Kyoto rats (WKYs) as the control strain, contrary to our works using WRs. WKYs might be an inappropriate control, considering that they display inactivity and depressive-like behavior (Overstreet, 2012). Moreover, the pharmacological validity of SHRs as a model for ADHD is controversial: studies show that the administration of the drugs used to treat ADHD, such as methylphenidate or amphetamine, is not able to improve SHRs ADHD-like behaviors, or even potentiates them (Amini et al., 2004; van den Bergh et al., 2006; Yang et al., 2006; Bizot et al., 2007; Barron et al., 2009; Calzavara et al., 2011). These concerns notwithstanding, a comorbidity between schizophrenia and adult ADHD has been described (Donev et al., 2011), and at high-risk young relatives of schizophrenia patients may display comorbid ADHD (Keshavan et al., 2003; Oner and Munir, 2005). In addition, schizophrenia patients or high-risk individuals with ADHD perform worse in neuropsychological tests, when compared to those without ADHD (Keshavan et al., 2003; Oner and Munir, 2005; Donev et al., 2011). Of relevance for this work, diagnosis of ADHD in peri-adolescence is associated with the emergence of schizophrenia symptomatology later in life (Kim-Cohen et al., 2003; Hennig et al., 2017). In this scenario, our data may also indicate that CBD displays a preventive potential for high-risk individuals with ADHD as a comorbidity or for those that develop schizophrenia and display a previous history of ADHD.

Increased locomotor activity is linked to augmented dopamine transmission in the mesolimbic system, and therefore is used to model the positive symptoms of schizophrenia (Lipska and Weinberger, 2000). Peripubertal administration of haloperidol, clozapine, risperidone or fluoxetine prevents the emergence of increased locomotor activity after the administration of psychostimulant drugs in the poly I:C model (Piontkewitz et al., 2009, 2011; Meyer et al., 2010), suggesting that these compounds could prevent the positive symptoms. Nevertheless, some of these early treatments had detrimental effects on the response to psychostimulant drugs of control animals (Meyer et al., 2010; Piontkewitz et al., 2011).

Contextual fear conditioning is a memory task with an emotional component. In the SHR strain, the contextual fear conditioning deficit is specifically reversed by the administration of antipsychotic drugs (Calzavara et al., 2009), being suggested as a model for the cognitive symptoms of schizophrenia. Contextual fear conditioning deficits are also seen in other animal models for schizophrenia (Duffy et al., 2010; Gill et al., 2017; Latusz et al., 2017). Our findings therefore point to beneficial effects of CBD on the cognitive impairments and are in accordance with a recent work showing that prolonged treatment with CBD improves the deficits on recognition and working memory in the poly I:C model (Osborne et al., 2017). It is noteworthy that Osborne et al. (2017) performed the treatment with CBD in a later stage (late adolescence/adulthood) and assessed the behaviors during treatment. Regarding preventive treatments, administration of antipsychotic drugs are able to prevent deficit on latent inhibition, a cognitive abnormality, in the poly I:C model (Piontkewitz et al., 2009, 2011; Meyer et al., 2010). On the other hand, adolescent administration of olanzapine impairs working memory in rats (Milstein et al., 2013).

PPI is a task used to assess the functioning of sensorimotor gating – a physiological process that filters sensory information, preventing overload and cognitive fragmentation (Powell et al., 2012). PPI deficits are seen in schizophrenia patients (Braff et al., 2001) and in several animal models for this disorder (Swerdlow et al., 2008). Data show that PPI deficits are associated with impaired functional status and with the presence of positive symptoms (Swerdlow et al., 2006; Kumari et al., 2008). Peripubertal administration of clozapine, but not haloperidol, prevents the emergence of PPI impairments in the poly I:C model (Meyer et al., 2010). In fact, early treatment with haloperidol promotes PPI deficits in the control offspring (Meyer et al., 2010). Here, we demonstrate, for the first time, that peripubertal treatment with CBD improves PPI deficits in an animal model for schizophrenia, without having detrimental effects on control animals.

Peripubertal treatment with CBD did not improve the reduced social interaction displayed by the SHRs. Indeed, while acute administration of CBD restores SHRs’ deficits on PPI and on contextual fear conditioning (Levin et al., 2012, 2014), it does not increase social interaction (Almeida et al., 2013). Beneficial effects of prolonged treatment with CBD on social interaction are seen in the poly I:C model (Osborne et al., 2017), however, in the presence of CBD administered during late adolescence/adulthood and using a higher dose (10 mg/kg, twice daily). The doses used here were chosen based on our previous work showing preventive effect (CBD was administered from days 30–60 and the beneficial effect on behavior was observed in adulthood) of 1 mg/kg CBD in the poly I:C model (Peres et al., 2016a). We chose to evaluate here a range of doses, considering that CBD effects usually follow an inverted U-shaped dose-response curve (Guimaraes et al., 1990; Nazario et al., 2015). In accordance, all beneficial effects of CBD were seen at the lowest dose (0.5 mg/kg). Nonetheless, we cannot disregard that using a higher dose of CBD could result on improvement of the social interaction deficit, as seen by Osborne et al. (2017).

In view of the low conversion rate and the risks of a peripubertal treatment, the search for preventive approaches must consider their safety. Peripubertal treatment with CBD increased social interaction as well as contextual fear conditioning response in WR control animals. Both behaviors can be modulated in this strain by acute administration of anxiolytic or anxiogenic compounds (File and Seth, 2003; Calzavara et al., 2009). Having in order that CBD also presents an anxiolytic profile, its long-term effect on WR strain (observed in the absence of the drug – 1 month after the end of the treatment) could be the result of a modification in anxiety levels. Nevertheless, social interaction and fear conditioning respond on opposite directions to anxiety drugs (contrary to what is seen here). It must be highlighted that CBD did not modify any other behavior in the control strain, indicating that it does not promote detrimental behavioral side effects. These data point to a safe use of chronic treatment with CBD during peri-adolescence in the individuals that will not develop schizophrenia in the adulthood.

It could be argued that a long-term modulation of anxiety by CBD might be related to its beneficial effects seen in the behavioral abnormalities displayed by the SHR strain. Nevertheless, the fear conditioning deficit of SHRs is improved by antipsychotic but not by anxiolytic or anxiogenic drugs (Calzavara et al., 2009). Conversely, anxiolytic drugs increase social interaction, an effect not seen in the present study. Additionally, PPI does not seem to be modulate by acute, chronic or deprivation of treatment with benzodiazepines (Rigdon and Viik, 1991; De Ross et al., 2008).

Still concerning the safety of a long-term treatment with CBD during peri-adolescence, we performed a screening of potential metabolic and motor side effects. Previous studies show that CBD does not modify physiological parameters, nor affects embryonic development or vitality of non-tumor cell lines (Bergamaschi et al., 2011; Iffland and Grotenhermen, 2017). CBD seems to be well tolerated, with the most reported side effects being changes on appetite, diarrhea, and tiredness (Bergamaschi et al., 2011; Iffland and Grotenhermen, 2017; Boggs et al., 2018; McGuire et al., 2018). Moreover, CBD has been reported to display a safe, or even beneficial, profile for the development of motor side effects (Peres et al., 2018).

Accordingly, here CBD did not alter animals’ body weight gain, serum levels of triglycerides or glycemia. Also, catalepsy and oral movements – motor side effects commonly seen with chronic antipsychotic use – were not induced. This is the first study to demonstrate that prolonged treatment with CBD during development does not promote detrimental motor or metabolic side effects in rodents. However, further studies are needed to warrant the safety of a treatment with CBD during a young age. It must be also considered that CBD can inactivate or induce cytochrome P450 isozymes, depending on the drug regimen, which may lead to important drug interactions (Bergamaschi et al., 2011; Iffland and Grotenhermen, 2017).

With respect to the antipsychotic profile of CBD in the SHR model, our previous works show that CBD restores the SHRs impairments in contextual fear conditioning and PPI (Levin et al., 2012, 2014), but does not modify SHRs locomotor activity or social interaction (Almeida et al., 2013). These data indicate that acute administration of CBD on adulthood ameliorates only SHRs cognitive deficits. In this respect, CBD differs from both typical and atypical antipsychotic drugs. Here, peripubertal treatment with CBD displayed beneficial effects on a broader spectrum of behaviors, considering that in addition to restore/prevent the emergence of cognitive impairments, CBD prevented hyperlocomotion. Concerning side effects, neither the motor nor the metabolic alterations – related to the chronic use of typical and atypical antipsychotics, respectively – were seen.

Cannabidiol treatment began at 30 days of age, when SHRs already display diminished social interaction, but do not display increased locomotor activity or impaired PPI (Niigaki et al., unpublished). Therefore, CBD was able to halt the emergence of hyperlocomotion and PPI deficits in the SHR strain. The underlying mechanisms whereby CBD exerts these preventive beneficial effects are likely multifaceted, given that CBD presents several molecular targets (Ibeas Bih et al., 2015). Our data suggest involvement of the serotoninergic system. Here, treatment with CBD increased the ratio 5-HIAA/serotonin on PND 61 and enhanced 5-HIAA levels on PND 90 in prefrontal cortex, suggesting an increased turnover of serotonin. In accordance, Linge et al. (2016) demonstrated that treatment with CBD for 14 days increases the serotonin release in the ventral-medial prefrontal cortex after a challenge dose of CBD. This effect might be related to the 5-HT_1A_ agonism proposed for CBD (Russo et al., 2005) because the 5-HT_1A_ antagonist WAY100635 prevents the increased serotonin release induced by acute administration of CBD (Linge et al., 2016). Interestingly, the administration of WAY100635 attenuates the increase in prefrontal cortex dopamine levels induced by atypical antipsychotic drugs with either low or high affinity for 5-HT_1A_ (Ichikawa et al., 2001, 2002). This increase in prefrontal dopamine levels is suggested to be relevant to the beneficial effects of these agents (McCreary and Newman-Tancredi, 2015). As a future perspective, investigating the role of 5-HT_1A_ receptors on the preventive effects of CBD by using 5-HT_1A_ antagonists would be of great relevance.

It must be noticed that the beneficial behavioral effects of CBD were observed 1 month after the interruption of treatment. The development of schizophrenia is strongly associated with changes in the neurodevelopmental course (Piper et al., 2012). Therefore, the preventive effects of CBD observed here are probably the results of a beneficial action on the neurodevelopmental process. In accordance, multiple studies describe CBD’s antioxidant, anti-inflammatory and neuroprotective actions. In pigs, CBD prevents the neuronal damage and the increases in excitotoxicity, oxidative stress and inflammation induced by a hypoxic-ischemic brain injury protocol (Pazos et al., 2013). The antioxidant effects of CBD are also seen in rat models of binge alcohol consumption, sepsis, epilepsy, Huntington’s disease, Parkinson’s disease, and mania (Hamelink et al., 2005; Cassol et al., 2010; Campos et al., 2017). CBD has also been reported to increase (Magen et al., 2010; Reus et al., 2011; Barichello et al., 2012; Campos et al., 2015; Mori et al., 2017; Sales et al., 2018) or decrease (ElBatsh et al., 2012) BDNF levels in rodents’ brains. Here, prolonged peripubertal treatment with CBD did not modify the BDNF levels in prefrontal cortex or striatum. In agreement with our findings, other authors report no changes on BDNF levels in prefrontal cortex (Reus et al., 2011; Campos et al., 2012, 2015) or striatum (ElBatsh et al., 2012) after repeated treatment with CBD (7 or 14 days). Moreover, in some studies CBD-induced alterations in BDNF levels are not associated with its behavioral effect (Magen et al., 2010; Reus et al., 2011). The search for neuroprotective targets other than BDNF that might underlie the beneficial CBD’s preventive effect is promising and currently under investigation by our group.

Another mechanism of action that might be involved in CBD’s preventive effects is the increase in the levels of endocannabinoids. Data suggest that anandamide, the main endocannabinoid, may display a protective role in schizophrenia: anandamide levels are inversely correlated with the psychotic symptoms in patients (Giuffrida et al., 2004). Moreover, Leweke et al. (2012) reported that treatment with CBD in schizophrenia patients results in a significant clinical improvement, which is positively associated with increase in anandamide levels. As a future perspective, we aim to quantify anandamide levels on brain regions of WRs and SHRs treated with CBD during peri-adolescence.

One important limitation of our work is that we evaluated the preventive effects of CBD only in male animals. This choice was based on the fact that the behavioral and pharmacological validation of the SHR strain as an animal model for schizophrenia was performed only with males (Calzavara et al., 2009, 2011; Levin et al., 2011). Considering that, in humans, females display a late age of schizophrenia onset (Donoghue et al., 2014), seem to require lower doses of antipsychotic drugs and to display higher incidence of side effects (Lange et al., 2017), the evaluation of pharmacological strategies in females is of extreme relevance. We are currently starting a behavioral and pharmacological characterization of adult SHR females to extend the validation of the model to female rats and allow future investigations.

In summary, the present study reinforces and extends the beneficial and safe preventive effects of peripubertal treatment with CBD on halting the emergence of behavioral abnormalities that mimic the positive and cognitive symptoms of schizophrenia. The translation of the present data to the clinical context indicates the possibility of diminishing the severe suffering of individuals that develop schizophrenia. Strengthening the relevance of our findings, while the positive symptoms of schizophrenia are devastating but treatable by antipsychotic drugs (with all the risk of developing significant side effects), the cognitive deficits associated with the disorder do not benefit from any current clinical treatment. Our innovative findings further support the notion that CBD has neuroprotective effects and may be a potential compound to prevent schizophrenia. Future double-blind, long-term, controlled clinical trials with large samples of people at high risk for psychosis would be necessary and opportune to further investigate these possibilities.

## Author Contributions

FP, AZ, JH, JC, and VCA designed the study. FP, MD, RL, MS, VA, AV, and CS conducted the experiments, statistical analysis, and managed the literature search. FP and VCA wrote the first draft of the manuscript. All authors contributed to approved the final manuscript.

## Conflict of Interest Statement

JH, AZ, and JC are co-inventors (Mechoulam R, JC, Guimaraes FS, AZ, JH, Breuer A) of the patent “Fluorinated CBD compounds, compositions and uses thereof. Pub. No.: WO/2014/108899. International Application No.: PCT/IL2014/050023”; Def. US no. Reg. 62193296; 29/07/2015; INPI em 19/08/2015 (BR1120150164927). University of São Paulo licensed it to Phytecs Pharm (Resolução USP No. 15.1.130002.1.1). University of São Paulo has an agreement with Prati-Donaduzzi (Toledo, Brazil): “Desenvolvimento de um produto farmacêutico contendo canabidiol sintético e comprovação de sua segurança e eficácia terapêutica na epilepsia, esquizofrenia, doença de Parkinson e transtornos de ansiedade.” JC and JH received a travel support from BSPG-Pharm. AZ, JH, and JC are medical advisors of BSPG-Pharm (UK). The remaining authors declare that the research was conducted in the absence of any commercial or financial relationships that could be construed as a potential conflict of interest.
